# TPx Protein of *Cysticercus cellulosae* Regulates Macrophage M2 Polarization via the cGMP-PKG Signaling Pathway

**DOI:** 10.3390/pathogens15080843

**Published:** 2026-08-13

**Authors:** Haiting Xiong, Xue Li, Haojun Cai, Qianqian Mu, Biying Zhou

**Affiliations:** Department of Parasitology, School of Basic Medical Sciences, Zunyi Medical University, Zunyi 563000, China; xht15575261045@163.com (H.X.); 15223811924@163.com (X.L.); 15672613277@163.com (H.C.); 18061503055@163.com (Q.M.)

**Keywords:** *Cysticercus cellulosae*, Thioredoxin peroxidase, cGMP-PKG signaling pathway, macrophage polarization

## Abstract

Cysticercosis, caused by the larval stage of *Taenia solium* (*Cysticercus cellulosae*), is a neglected tropical disease threatening public health. Thioredoxin peroxidase (TPx) is a key antioxidant protein secreted by the parasite, but its role in macrophage polarization remains unclear. In this study, THP-1-derived macrophages were treated with TPx protein for 24 h and 48 h. Flow cytometry, reverse transcription-quantitative polymerase chain reaction (RT-qPCR), and Western blot were employed to assess reactive oxygen species (ROS) levels, M1/M2 cell proportions, mRNA expression of tumor necrosis factor-alpha (*TNF-α*) and interleukin-10 (*IL-10*), and protein expression of inducible nitric oxide synthase (iNOS) and arginase-1 (Arg-1). Transcriptome sequencing was performed to screen for signaling pathways, and enzyme-linked immunosorbent assay (ELISA) was subsequently used to measure cGMP levels. The PKG inhibitor KT-5823 was used for functional validation. The results show that TPx significantly increased the proportion of M1 macrophages from 4.58% to 9.24% at 24 h, and promoted M2 macrophages from 4.12% to 6.87% at 48 h, while ROS levels decreased to 0.80-fold at 48 h (*p* < 0.05). RT-qPCR revealed that TPx markedly upregulated *TNF-α* (1.62-fold) at 24 h and *IL-10* (1.52-fold) at 48 h (*p* < 0.05). Western blot showed that TPx increased iNOS expression by 2.55-fold at 24 h and Arg-1 expression by 2.01-fold at 48 h. KEGG analysis revealed upregulation of the cGMP-PKG pathway at 48 h, with notably increased cGMP content (1.48-fold) and PKG expression (1.86-fold) (*p* < 0.05). Furthermore, KT-5823 pretreatment effectively reversed TPx-induced Arg-1 upregulation (from 1.66-fold to 1.02-fold, *p* < 0.05). These findings demonstrate that *Cysticercus cellulosae* TPx induces M1 polarization at 24 h and promotes M2 polarization at 48 h through activation of the cGMP-PKG signaling pathway, thereby facilitating immune evasion.

## 1. Introduction

*Taenia solium* is an important foodborne zoonotic parasite [1]. Its larval stage, *Cysticercus cellulosae*, can parasitize humans or pigs, causing cysticercosis. The disease is globally distributed, mainly prevalent in economically underdeveloped regions such as Latin America, Asia and Africa [2]. The parasite can invade the brain, subcutaneous tissues, muscles, and other sites in humans. Among these, neurocysticercosis (NCC) is relatively severe, capable of causing central nervous system symptoms such as epileptic seizures, elevated intracranial pressure, and neuropsychiatric disorders, and can lead to death in severe cases [3,4]. Current treatment options remain limited [5], and the immunopathogenesis of the disease is not yet fully understood, severely hindering the development of diagnostic targets and vaccines.

Macrophages, derived from monocytes, are essential components of the host immune system and can initiate adaptive immune responses through antigen presentation during parasitic infections [6,7]. Moreover, macrophages exhibit high plasticity and can polarize into classically activated (M1) or alternatively activated (M2) phenotypes in response to different microenvironmental stimuli. M1 macrophages secrete pro-inflammatory cytokines such as tumor necrosis factor-alpha (TNF-α) and interleukin-12 (IL-12), and highly express molecules such as inducible nitric oxide synthase (iNOS) and CD86, primarily participating in host anti-infective and anti-tumor immunity. M2 macrophages secrete anti-inflammatory cytokines such as IL-10 and transforming growth factor-β (TGF-β), and highly express molecules such as arginase-1 (Arg-1) and macrophage mannose receptor (CD206), playing important roles mainly in tissue repair, immune regulation, and tumor progression [8,9]. Parasitic infections can induce a host Th2-type immune response, promoting the secretion of cytokines such as IL-4 and IL-13, which in turn drive macrophage M2 polarization and establish an immunosuppressive microenvironment [10,11]. Concurrently, M2 macrophages highly express Arg-1, which consumes L-arginine to inhibit iNOS activity and NO production, thereby weakening the parasiticidal functions of M1 macrophages. Furthermore, the metabolite proline derived from Arg-1-mediated arginine metabolism contributes to tissue repair, facilitating long-term parasite survival [12,13]. Thus, macrophage M2 polarization is of great significance in parasite immune evasion.

Parasite excretory–secretory antigens (ESAs) can modulate host immune cells to facilitate immune regulation, thereby promoting long-term parasite survival [14,15]. Studies have demonstrated that *Taenia solium* ESA can induce macrophage M2 polarization [16]. Furthermore, our group previously found that *Cysticercus cellulosae* ESA predominantly induced a Th2-type immune response in piglets [17], indirectly suggesting its potential to induce macrophage M2 polarization. However, the key functional protein(s) within *Cysticercus cellulosae* ESA responsible for this effect have not yet been fully elucidated.

Thioredoxin peroxidase (TPx), a member of the peroxiredoxin family, is widely present in the ESA of parasites. It functions to scavenge reactive oxygen species (ROS), thereby maintaining intracellular redox homeostasis [18,19]. Moreover, high ROS levels promote macrophage M1 polarization, whereas low ROS levels favor M2 polarization [20]. Notably, TPx protein is also extensively involved in the immunoregulatory processes of parasites. Previous studies have confirmed that TPx proteins secreted by parasites such as *Trichinella spiralis*, *Fasciola hepatica*, *Schistosoma japonicum*, *Toxoplasma gondii*, *Echinococcus multilocularis*, and *Echinococcus granulosus* can induce macrophage M2 polarization [21,22,23,24,25,26,27]. However, whether *Cysticercus cellulosae* TPx protein is involved in regulating macrophage polarization remains unexplored.

Based on the above findings, this study aims to employ a THP-1-derived macrophage model to investigate the effect of *Cysticercus cellulosae* TPx on macrophage polarization, and to further explore the role of the cGMP-PKG signaling pathway in TPx-induced macrophage M2 polarization using transcriptomic analysis and Western blotting. This study provides a new experimental basis for elucidating the immunopathogenesis of cysticercosis.

## 2. Materials and Methods

### 2.1. Preparation of Cysticercus cellulosae TPx Protein

The TPx gene was synthesized and cloned into the pcDNA3.4 vector. The recombinant plasmid was verified by restriction enzyme digestion and DNA sequencing and then transfected into Expi293 cells. The expressed TPx protein was purified using Ni^2+^ affinity chromatography (endotoxin level < 10 EU/mg), identified by SDS-PAGE and Western blot, quantified by BCA protein assay kit (Nanjing Jiancheng Bioengineering Institute, Nanjing, China), and then aliquoted and stored at −80 °C.

### 2.2. Cell Culture, Differentiation, and Treatment

THP-1 cells were purchased from the Cell Bank of the Chinese Academy of Sciences (Shanghai, China) and cultured in RPMI 1640 medium (Meilunbio, Dalian, China) supplemented with 10% fetal bovine serum (FBS) (Absin, Shanghai, China) and 1% penicillin-streptomycin (Solarbio, Beijing, China) at 37 °C in a 5% CO_2_ incubator (Thermo Fisher Scientific, Waltham, MA, USA). The medium was replaced every 2–3 days. Cells were seeded at a density of 1 × 10^6^ cells/mL and induced to differentiate into adherent macrophages by stimulation with 100 ng/mL phorbol 12-myristate 13-acetate (PMA) (Solarbio, Beijing, China) for 48 h (see Supplementary Figure S1) for subsequent experiments.

To investigate the effect of TPx protein on macrophage polarization, macrophages were divided into control and TPx-treated groups (500 ng/mL) and cultured for 24 h and 48 h, respectively. To verify the involvement of the cGMP-PKG signaling pathway in TPx-induced M2 polarization, additional groups were established: control, TPx, KT-5823, and TPx + KT-5823 groups. KT-5823 (MedChemExpress, Monmouth Junction, NJ, USA) was dissolved in dimethyl sulfoxide (DMSO) (Solarbio, Beijing, China). The KT-5823 and TPx + KT-5823 groups were pretreated with 1 μM KT-5823 for 24 h (Supplementary Figure S8), followed by incubation with 500 ng/mL TPx protein for an additional 48 h in the TPx and TPx + KT-5823 groups.

### 2.3. Detection of Intracellular ROS Levels in Macrophages

Macrophages treated with *Cysticercus cellulosae* TPx protein for 24 h or 48 h were collected and incubated with 2′,7′-dichlorodihydrofluorescein diacetate (DCFH-DA) probe (ROS Assay Kit, Beyotime, Shanghai, China) at a dilution of 1:600,000 in a 37 °C cell culture incubator for 30 min in the dark. After washing three times with phosphate-buffered saline (PBS) (Meilunbio, Dalian, China), 3 mL of PBS was added, and cells were scraped with a cell scraper and filtered through a 100 μm cell strainer. The filtrate was centrifuged at 1500 rpm for 5 min, the supernatant was discarded, and the cells were resuspended in 300 μL of PBS. Fluorescence intensity was detected by Northern Lights flow cytometry (Cytek Biosciences, Fremont, CA, USA), and the results are expressed as mean fluorescence intensity.

### 2.4. Flow Cytometry Analysis

Cells from each group were collected and digested with 1 mL of 0.25% trypsin (Servicebio, Wuhan, China), and the digestion was terminated with complete medium. The cell suspension was filtered through a 100 μm cell strainer and centrifuged at 1000 rpm for 5 min, and the supernatant was discarded. The cells were resuspended in 200 μL of PBS and incubated with 1 μL of CD11b-FITC (BD Pharmingen, San Diego, CA, USA), 5 μL of CD86-PE (BD Pharmingen, San Diego, CA, USA), and 5 μL of CD206-APC (BD Pharmingen, San Diego, CA, USA) antibodies at 4 °C for 30 min in the dark. After incubation, 1.5 mL of PBS was added, and the cells were centrifuged at 1200 rpm for 10 min. The supernatant was discarded, and the cells were resuspended in 300 μL of PBS. The proportions of CD11b^+^CD86^+^ M1 and CD11b^+^CD206^+^ M2 macrophages were detected by BD FACSAria III flow cytometry (BD Biosciences, San Jose, CA, USA). The antibody volumes used above were determined according to the manufacturer’s instructions and laboratory routine practice.

### 2.5. RT-qPCR Analysis

Total RNA was extracted from each group of cells using RNAiso Plus reagent (TaKaRa, Kusatsu, Japan). RNA concentration was determined by spectrophotometry, and 500 ng of RNA was reverse-transcribed into cDNA using the PrimeScript™ RT Reagent Kit (TaKaRa, Kusatsu, Japan) on a C1000 Touch Thermal Cycler (Bio-Rad, Hercules, CA, USA) according to the manufacturer’s instructions. Real-time PCR was performed using TB Green^®^ Premix Ex Taq™ II (TaKaRa, Kusatsu, Japan) on a Bio-Rad CFX96 system (Bio-Rad, Hercules, CA, USA). The relative expression levels of target genes were calculated using the 2^−ᐃᐃCt^ method, with *GAPDH* serving as the internal reference (primer sequences are provided in Supplementary Table S1).

### 2.6. Western Blot Analysis

Cells from each group were collected and lysed in radioimmunoprecipitation assay (RIPA) lysis buffer (Solarbio, Beijing, China) containing 1% phenylmethanesulfonyl fluoride (PMSF) (Solarbio, Beijing, China) and 1% phosphatase inhibitor cocktail (Meilunbio, Dalian, China) on ice for 30 min. The lysates were centrifuged at 13,000 rpm for 15 min at 4 °C, and the supernatant was collected as total protein. Protein concentration was measured by BCA assay, and equal amounts of protein were separated by 10% or 12.5% SDS-PAGE and transferred onto 0.45 μm PVDF membranes. The membranes were blocked with 5% non-fat dry milk in Tris-buffered saline containing 0.1% Tween-20 (TBST) at room temperature for 2.5 h. After washing three times with TBST, the membranes were incubated overnight at 4 °C with primary antibodies against iNOS (Proteintech, Wuhan, China), Arg-1 (Absin, Shanghai, China), PKG (Absin, Shanghai, China), NF-κB p65 (Proteintech, Wuhan, China), p-NF-κB p65 (Absin, Shanghai, China), GAPDH (Proteintech, Wuhan, China), and β-actin (Proteintech, Wuhan, China). Following three washes with TBST, the membranes were incubated with HRP-conjugated secondary antibodies (Absin, Shanghai, China) at room temperature for 1 h. After another three washes with TBST, the protein bands were visualized using enhanced chemiluminescence (ECL) detection reagent (Meilunbio, Dalian, China) and imaged on a Bio-Rad ChemiDoc MP Imaging System (Bio-Rad, Hercules, CA, USA). Band intensities were analyzed using Image Lab 6.1 software, normalized to GAPDH or β-actin as internal controls, and the relative expression levels of each experimental group were calculated after normalization to the mean value of the control group.

### 2.7. Transcriptome Sequencing and Bioinformatics Analysis

Total RNA was extracted from macrophages treated with *Cysticercus cellulosae* TPx protein for 24 h and 48 h, and sent to Shanghai Majorbio Bio-pharm Technology Co., Ltd. (Shanghai, China) for quality control. After RNA quality was confirmed, cDNA library construction and quality assessment were performed, and high-throughput sequencing was carried out on qualified libraries. Gene expression levels were quantified as transcripts per million (TPM). Differentially expressed genes (DEGs) were screened using DESeq2 with the criteria of |log_2_fold change (FC)| ≥ 0.585 and *p* < 0.05, and the identified DEGs were subjected to Gene Ontology (GO) functional annotation, Kyoto Encyclopedia of Genes and Genomes (KEGG) pathway enrichment analysis and gene clustering analysis. In addition, Gene Set Enrichment Analysis (GSEA) was performed based on all detected genes.

### 2.8. ELISA Assay

Cells from each group were collected, washed three times with PBS, and resuspended in 1 mL PBS containing 1% PMSF. The cells were lysed by ice-bath ultrasonication using an ultrasonic cell disruptor (Scientz, Ningbo, China), and the lysates were centrifuged at 4 °C and 4000 rpm for 10 min. The resulting supernatants were collected for further analysis. The cGMP content in the supernatants was measured using a cGMP ELISA kit (Baipeng Biotechnology, Chengdu, China), strictly following the manufacturer’s instructions. The absorbance was read at 450 nm, and the cGMP concentration was calculated using a standard curve constructed by four-parameter logistic curve fitting (R^2^ > 0.99). The results were normalized to the total protein concentration and expressed as pg/mg protein.

### 2.9. Statistical Analysis

All experimental data are presented as mean ± standard deviation (SD). RT-qPCR was performed with *n* = 4, while all other experiments were conducted with *n* = 3. Statistical analyses were performed using SPSS 29.0 software. For continuous data, after testing for normality and homogeneity of variance, comparisons between two groups were performed using the independent samples *t*-test, while comparisons among multiple groups were conducted by one-way analysis of variance followed by the least significant difference *t*-test for pairwise comparisons. For data that did not meet the assumptions, the Mann–Whitney U test was applied. A *p*-value *<* 0.05 was considered statistically significant.

## 3. Results

### 3.1. Effect of Cysticercus cellulosae TPx Protein on Intracellular Reactive Oxygen Species (ROS) Levels in Macrophages

**Figure 1:**
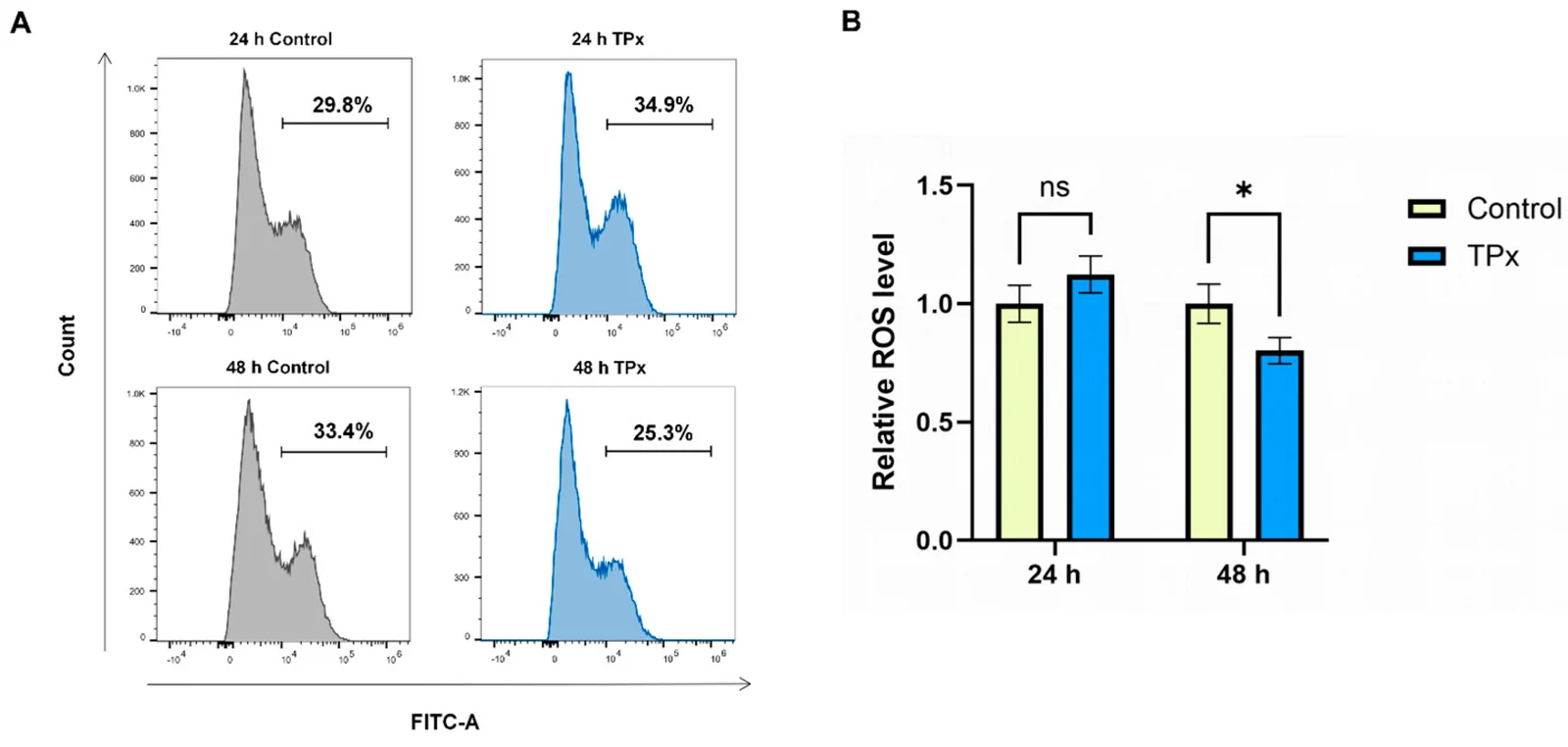
Effect of *Cysticercus cellulosae* TPx protein on intracellular ROS levels in macrophages. (**A**) Representative flow cytometry histograms of intracellular ROS levels in each group. (**B**) Statistical analysis of relative ROS levels. Data are presented as mean ± SD (*n* = 3). * indicates *p* < 0.05, ns indicates *p* > 0.05.

After 24 h of TPx protein treatment, no significant difference in the intracellular ROS level was observed in macrophages (*p* > 0.05); after 48 h of treatment, the intracellular ROS level was significantly decreased (*p* < 0.05) (Figure 1).

### 3.2. Effect of Cysticercus cellulosae TPx Protein on Macrophage Polarization

**Figure 2:**
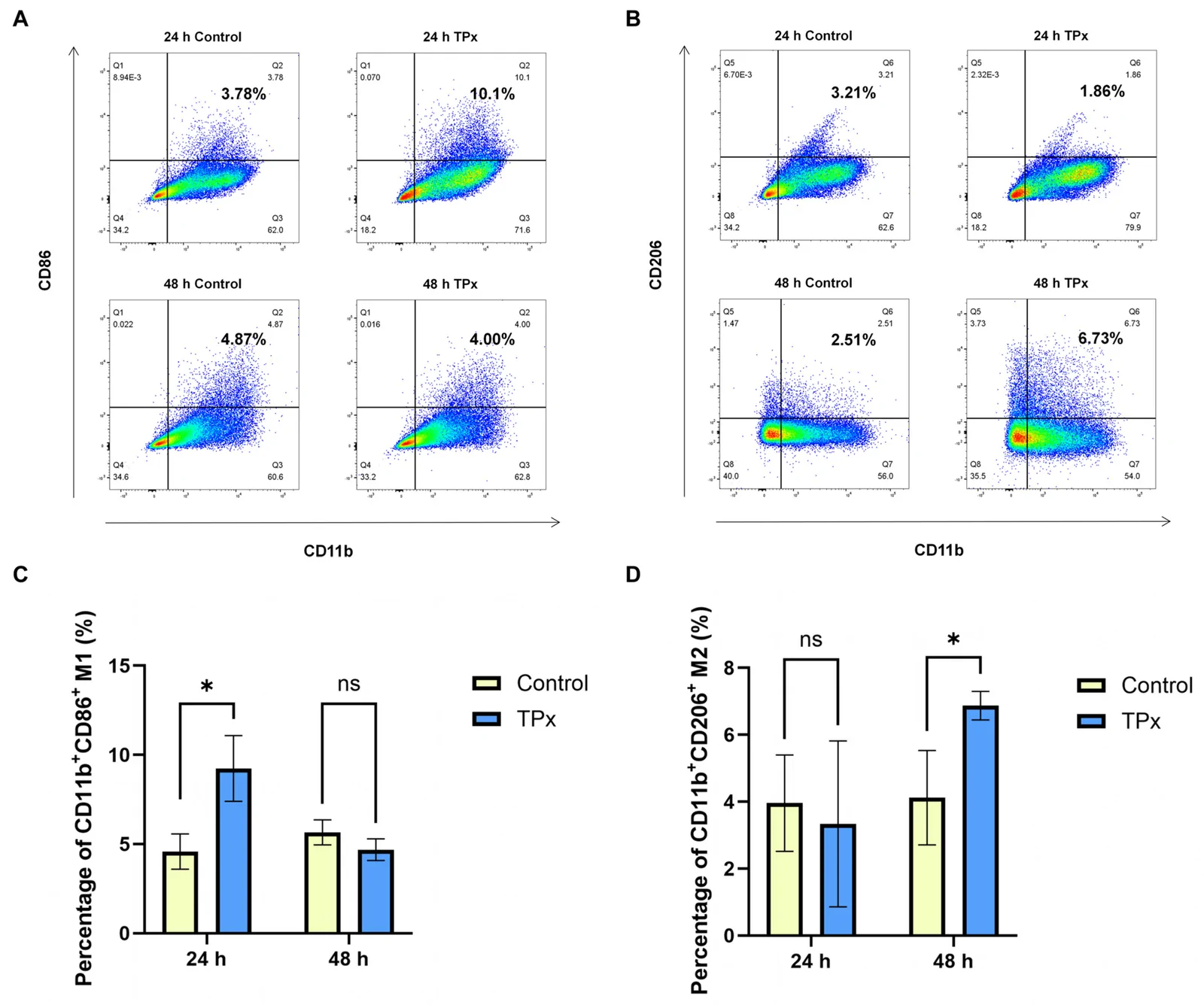
Effect of *Cysticercus cellulosae* TPx protein on macrophage polarization. (**A**) Representative flow cytometry scatter plots of M1 macrophages (CD11b^+^CD86^+^). (**B**) Representative flow cytometry scatter plots of M2 macrophages (CD11b^+^CD206^+^). (**C**) Quantification of the percentage of M1 macrophages. (**D**) Quantification of the percentage of M2 macrophages. Data are presented as mean ± SD (*n* = 3). * indicates *p* < 0.05, ns indicates *p* > 0.05.

After 24 h of TPx protein treatment, the proportion of M1 (CD11b^+^CD86^+^) macrophages was significantly increased (*p* < 0.05), while the proportion of M2 (CD11b^+^CD206^+^) macrophages showed no significant change (*p* > 0.05); after 48 h of TPx protein treatment, the proportion of M1 (CD11b^+^CD86^+^) macrophages showed no significant change (*p* > 0.05), whereas the proportion of M2 (CD11b^+^CD206^+^) macrophages was significantly increased (*p* < 0.05) (Figure 2).

### 3.3. Effect of Cysticercus cellulosae TPx Protein on Macrophage Polarization-Associated Molecules

**Figure 3:**
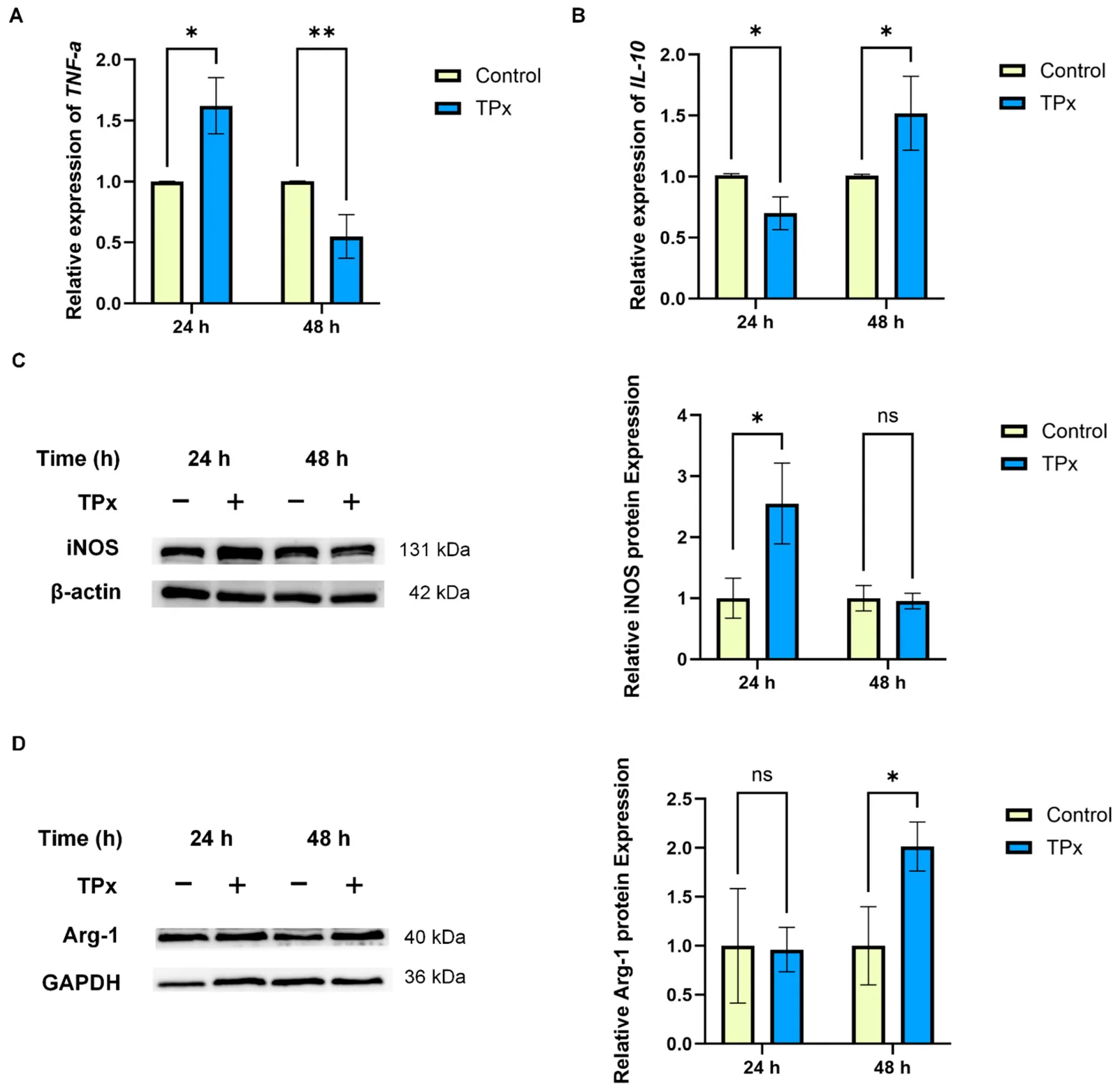
Effect of *Cysticercus cellulosae* TPx protein on macrophage polarization-associated molecules. (**A**) Relative mRNA expression of *TNF-α*. (**B**) Relative mRNA expression of *IL-10*. (**C**) Representative Western blot bands and quantification of iNOS protein expression. (**D**) Representative Western blot bands and quantification of Arg-1 protein expression. Data are presented as mean ± SD. For RT-qPCR (**A**,**B**), *n* = 4; for Western blot (**C**,**D**), *n* = 3. * indicates *p* < 0.05, ** indicates *p* < 0.01, ns indicates *p* > 0.05.

After 24 h of TPx protein treatment, the mRNA transcription level of *TNF-α* was significantly increased (*p* < 0.05), while that of *IL-10* was significantly decreased (*p* < 0.05) (Figure 3A,B); iNOS protein expression was significantly increased (*p* < 0.05), while Arg-1 protein expression showed no significant change (*p* > 0.05) (Figure 3C,D). After 48 h of TPx protein treatment, the mRNA transcription level of *TNF-α* was significantly decreased (*p* < 0.05), while that of *IL-10* was significantly increased (*p* < 0.05) (Figure 3A,B); iNOS protein expression showed no significant change (*p* > 0.05), whereas Arg-1 protein expression was significantly increased (*p* < 0.05) (Figure 3C,D).

### 3.4. Transcriptomic Analysis

**Figure 4:**
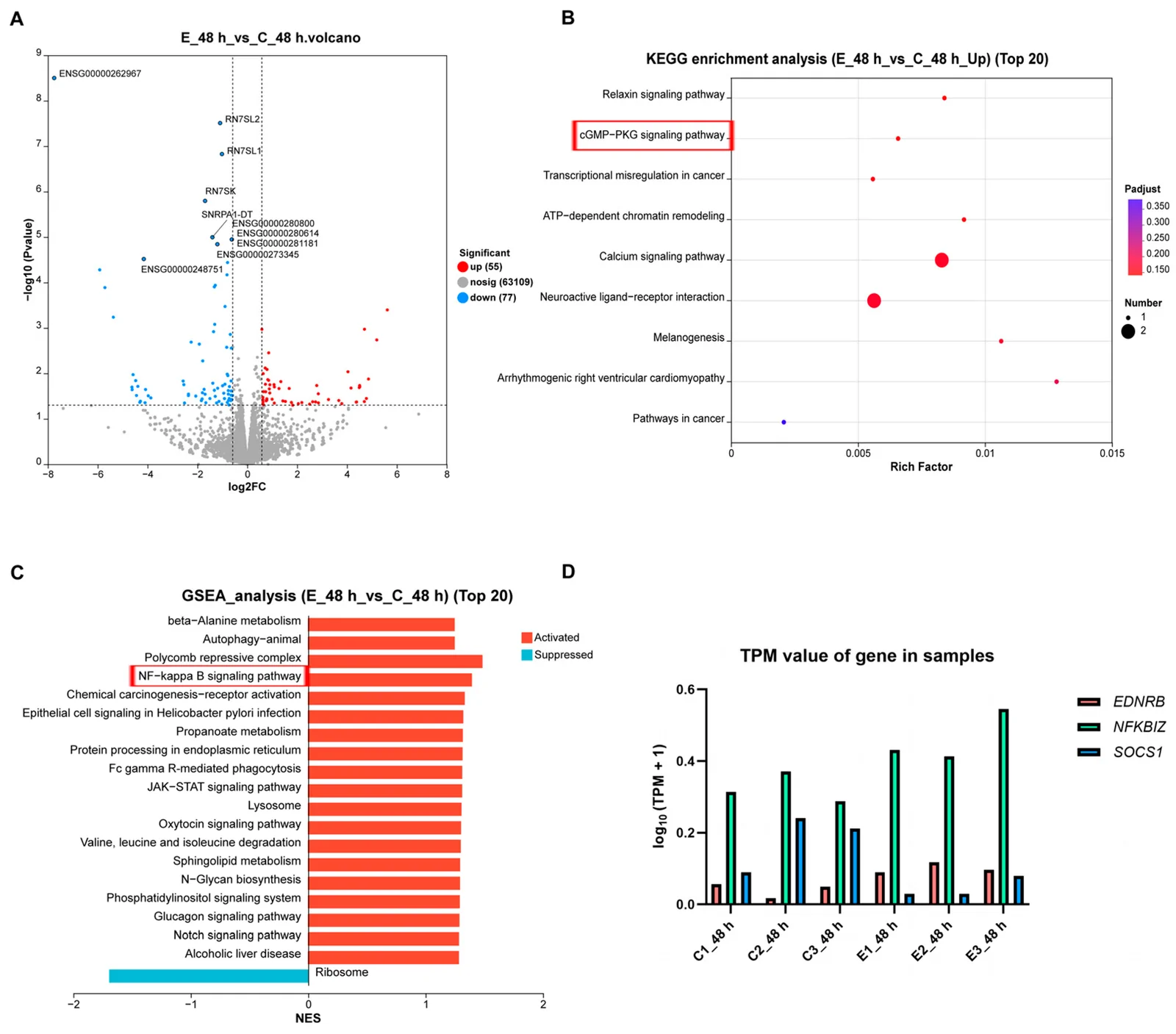
Transcriptomic analysis of macrophages treated with TPx protein for 48 h. (**A**) Volcano plot of differentially expressed genes. The x-axis represents log_2_fold change, and the y-axis represents the statistical significance. Blue, red, and gray dots indicate significantly downregulated, significantly upregulated, and non-significantly changed genes, respectively. Points closer to the sides and top represent greater differences. (**B**) KEGG enrichment bubble plot (upregulated). The x-axis represents the rich factor, and the y-axis lists the KEGG pathway names. A larger rich factor indicates a higher degree of pathway enrichment. Bubble size represents the number of enriched genes, and bubble color corresponds to different adjusted *p*-value ranges; redder color indicates stronger significance. (**C**) GSEA enrichment bar chart. The x-axis represents the normalized enrichment score (NES), and the y-axis lists the gene set names. “Activated” indicates that the corresponding gene set is ctivated, and “Suppressed” indicates that it is suppressed. (**D**) Gene clustering dot-line plot. C1_48 h, C2_48 h, and C3_48 h represent three replicates of the 48 h control group; E1_48 h, E2_48 h, and E3_48 h represent three replicates of the 48 h experimental group. The y-axis, log_10_(TPM + 1), represents the gene transcription level.

To explore the molecular mechanism underlying TPx-induced macrophage M2 polarization, transcriptome sequencing was performed on macrophages treated with TPx protein for 48 h. A total of 132 differentially expressed genes (DEGs) were identified, including 55 upregulated and 77 downregulated genes (Figure 4A). KEGG enrichment analysis revealed that signaling pathways such as cGMP-PKG were significantly upregulated (Figure 4B). GSEA indicated that signaling pathways including NF-κB were activated (Figure 4C). Furthermore, gene clustering analysis showed increasing trends in the transcription levels of *EDNRB* and *NFKBIZ*, and a decreasing trend in *SOCS1* transcription (Figure 4D). The remaining transcriptomic analysis results are provided in Supplementary Figures S2–S7.

### 3.5. Effect of TPx Protein on Molecules Related to the cGMP-PKG and NF-κB Signaling Pathways

**Figure 5:**
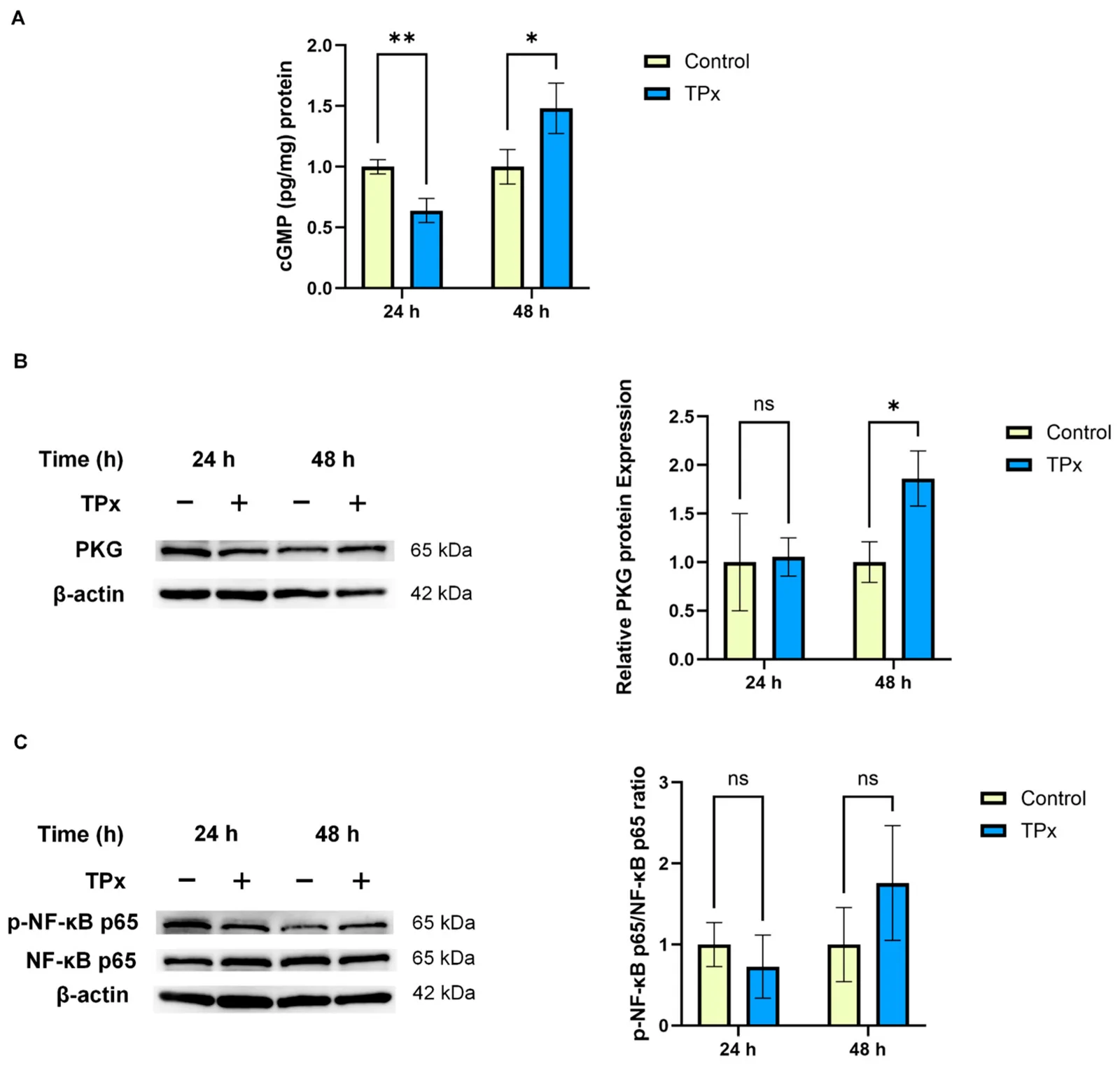
Effect of TPx protein on molecules related to the cGMP-PKG and NF-κB signaling pathways. (**A**) cGMP content detected by ELISA. (**B**) Representative Western blot bands and quantification of PKG protein expression. (**C**) Representative Western blot bands of p-NF-κB p65 and NF-κB p65, and quantification of the p-NF-κB p65/NF-κB p65 ratio. Data are presented as mean ± SD (*n* = 3). * indicates *p* < 0.05, ** indicates *p* < 0.01, ns indicates *p* > 0.05.

Based on the above transcriptomic analysis results, it was hypothesized that the cGMP-PKG and NF-κB signaling pathways might play important roles in *Cysticercus cellulosae* TPx protein-mediated regulation of macrophage M2 polarization. Therefore, the cGMP content and the protein expression levels of PKG, NF-κB p65, and p-NF-κB p65 were further examined. After 24 h of TPx protein treatment, the cGMP content was significantly decreased (*p* < 0.01), while the protein expression levels of PKG and p-NF-κB p65 both showed downward trends without significant differences (*p* > 0.05). After 48 h of TPx protein treatment, the cGMP content and PKG protein expression were significantly increased (*p* < 0.05), while the p-NF-κB p65 protein expression showed an upward trend without a significant difference (*p* > 0.05) (Figure 5).

### 3.6. Effect of KT-5823 on TPx Protein-Induced Macrophage M2 Polarization

**Figure 6:**
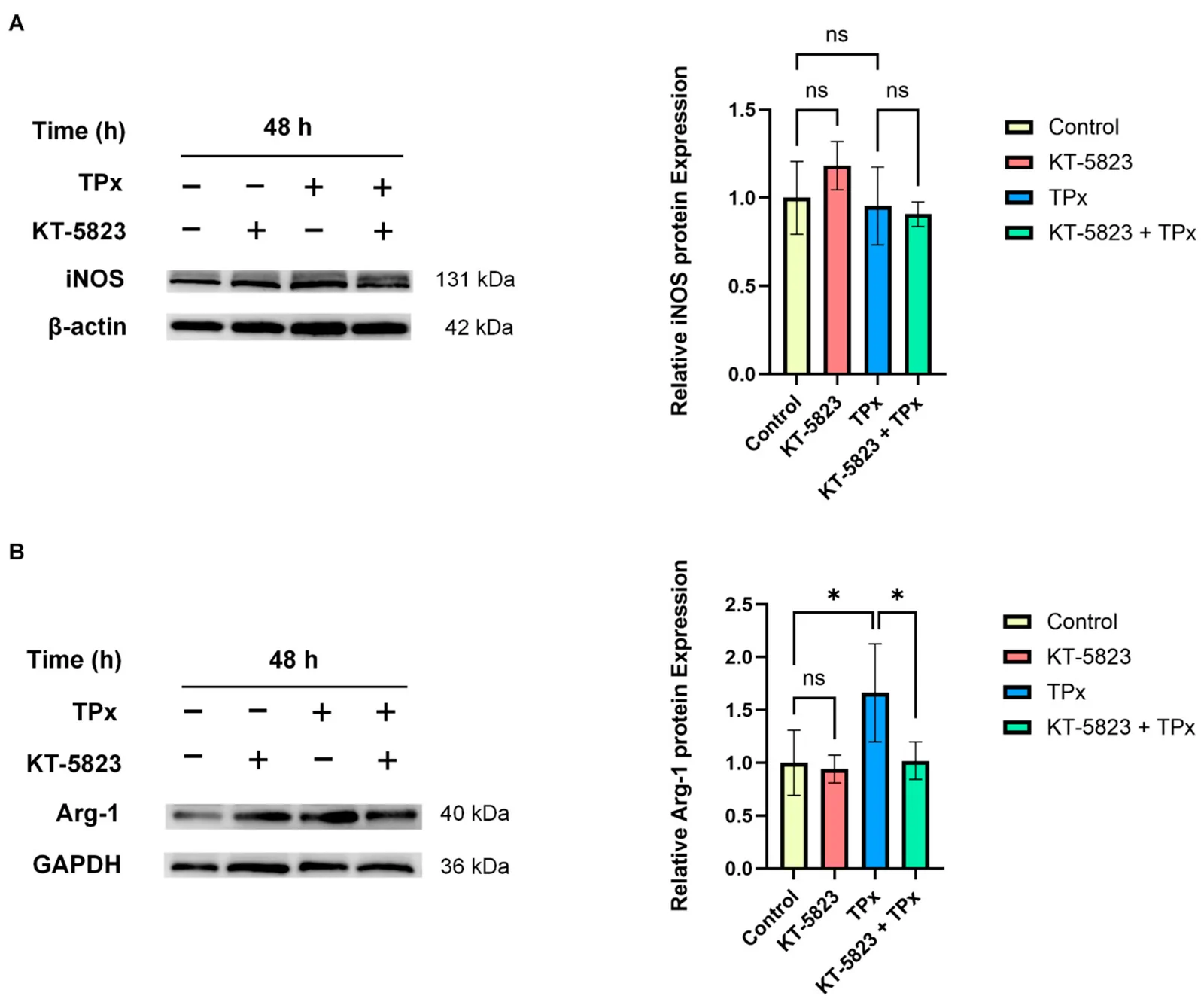
Effect of KT-5823 on TPx protein-induced macrophage M2 polarization. (**A**) Representative Western blot bands and quantification of iNOS protein expression. (**B**) Representative Western blot bands and quantification of Arg-1 protein expression. Data are presented as mean ± SD (*n* = 3). * indicates *p* < 0.05, ns indicates *p* > 0.05.

To investigate the role of the cGMP-PKG signaling pathway in TPx protein-induced macrophage polarization, the safe working concentration of the PKG inhibitor KT-5823 was first determined by CCK-8 assay (see Supplementary Figure S8), and 1 μM was selected for subsequent experiments. Macrophages were pretreated with 1 μM KT-5823 for 24 h, and then co-cultured with TPx protein for 48 h. Western blot results show that KT-5823 significantly inhibited the TPx-induced upregulation of Arg-1 protein expression (*p* < 0.05), while no significant effect on iNOS protein expression was observed (*p* > 0.05) (Figure 6).

## 4. Discussion

Cysticercosis remains a major public health problem, and the lack of an effective vaccine for humans is largely attributable to the complexity of its pathogenic mechanisms [28,29]. Macrophage polarization plays a critical role in parasite immune evasion, yet its regulatory mechanisms in cysticercosis have not been fully elucidated. In this study, we found that *Cysticercus cellulosae* TPx protein promoted M1 macrophage polarization at 24 h (indicated by significantly increased CD11b^+^CD86^+^ cells and elevated TNF-α and iNOS levels) and M2 polarization at 48 h (indicated by significantly increased CD11b^+^CD206^+^ cells and elevated IL-10 and Arg-1 levels). This temporal polarization pattern is consistent with that observed in infections by *Neospora caninum*, *Clonorchis sinensis*, *Echinococcus multilocularis*, *Schistosoma japonicum*, *Trichinella spiralis* and *Thelazia callipaeda* [30,31,32,33,34,35]. As a key functional molecule of M2 macrophages, Arg-1 can promote parasite survival and proliferation, facilitating the establishment of chronic infection [36,37]. Therefore, the M2 polarization mediated by TPx protein at 48 h may represent a key strategy employed by *Cysticercus cellulosae* to achieve host immune evasion.

Studies have shown that macrophage polarization is precisely regulated by multiple signaling pathways [38]. To explore the molecular mechanisms governing macrophage M2 polarization in cysticercosis, we performed transcriptome sequencing on macrophages treated with *Cysticercus cellulosae* TPx protein for 48 h. KEGG enrichment analysis revealed upregulation of the cyclic guanosine monophosphate–protein kinase G (cGMP-PKG) signaling pathway. This pathway depends on the second messenger cGMP to activate PKG and is extensively involved in immune regulation, playing a critical role particularly in macrophage polarization [39,40]. For instance, the alcohol dehydrogenase 1A (*ADH1A*) gene can influence human macrophage M1 polarization through the cGMP-PKG signaling pathway [41], and knockdown of VASP can promote M2 polarization and alleviate LPS-induced acute lung injury via the same pathway [42]. Furthermore, gene clustering analysis revealed that endothelin receptor type B (*EDNRB*) showed an upregulation trend after 48 h of TPx protein treatment. *EDNRB* is a G protein-coupled receptor that can activate the cGMP-PKG signaling pathway and is associated with the immunosuppressive function of macrophages [43,44]. These results suggest that the cGMP-PKG signaling pathway may play an important role in TPx-induced macrophage M2 polarization. Meanwhile, gene clustering analysis also revealed a decreasing trend in the transcription level of suppressor of cytokine signaling 1 (*SOCS1*). As a negative regulator of STAT6, previous studies have demonstrated that IL-4-induced Arg-1 gene transcription is significantly enhanced through a STAT6-dependent mechanism in macrophages from *SOCS1*^−/−^ mice [45]. This suggests that the downregulation of *SOCS1* may attenuate the negative feedback inhibition of STAT6, thereby promoting M2 polarization, which may serve as an auxiliary mechanism synergistically regulating M2 polarization with the cGMP-PKG signaling pathway; however, this hypothesis requires further investigation.

To verify whether the cGMP-PKG pathway was activated, we examined the relevant key molecules in this pathway. The results show that both the cGMP content and PKG protein expression were significantly increased, confirming that this signaling pathway was significantly activated after 48 h of TPx protein treatment. Notably, PKG can directly phosphorylate the NF-κB p65 subunit in T lymphocytes and also indirectly regulate its transcriptional activity [46]. Previous studies have shown that C-type natriuretic peptide can downregulate NF-κB p65 protein expression by activating the cGMP-PKG signaling pathway, thereby exerting anti-inflammatory effects in a rat model of epididymitis induced by Escherichia coli [47]. In the present study, GSEA analysis indicated that the NF-κB signaling pathway was activated; however, the protein expression of p-NF-κB p65 only showed an upward trend, which may be related to the fact that NF-κB, as an early transcription factor, had entered a negative feedback regulation stage by 48 h [48]. Furthermore, this study found that nuclear factor κB inhibitor ζ (*NFKBIZ*) exhibited an increasing trend after 48 h of TPx protein treatment. The inhibitor of κB ζ (IκBζ), encoded by *NFKBIZ*, preferentially binds to the NF-κB p50 subunit rather than p65 [49]. This is consistent with the activation trend of the NF-κB pathway, suggesting that *NFKBIZ* may participate in TPx protein-induced macrophage M2 polarization through a p50-dependent pathway.

To further validate the role of the cGMP-PKG signaling pathway in TPx protein-mediated regulation of macrophage M2 polarization, we pretreated macrophages with the PKG inhibitor KT-5823. The results show that KT-5823 alone had no significant effect on the protein expression of iNOS or Arg-1, suggesting that inhibition of PKG alone does not alter macrophage polarization status. However, KT-5823 pretreatment significantly blocked the TPx protein-induced upregulation of Arg-1 protein expression, while showing no significant effect on iNOS protein expression. These results indicate that activation of the cGMP-PKG signaling pathway is one of the key mechanisms underlying TPx protein-induced macrophage M2 polarization.

Nitric oxide (NO) is an upstream activator of the cGMP-PKG signaling pathway, which can promote cGMP production by activating soluble guanylyl cyclase (sGC), thereby activating PKG, and can also promote macrophage M1 polarization via VASP [50]. Moreover, the activation of the cGMP-PKG signaling pathway depends not only on NO but is also regulated by ROS. Studies have shown that ROS can induce NOS uncoupling to reduce NO production, oxidize sGC to decrease its sensitivity to NO, and directly inhibit PKG activity [51]. In the present study, ROS levels were significantly reduced and the cGMP-PKG pathway was activated in macrophages after 48 h of TPx protein treatment. This suggests that TPx protein may reduce ROS levels through its antioxidant function, thereby alleviating the above-mentioned inhibitory mechanisms, maintaining cGMP-PKG pathway activation, and promoting macrophage M2 polarization.

In summary, the results of this study demonstrate that after 24 h of TPx protein treatment, ROS levels showed an increasing trend, and macrophages polarized toward the M1 phenotype; whereas after 48 h of treatment, ROS levels were significantly reduced, and macrophages polarized toward the M2 phenotype. Transcriptomic analysis revealed that the cGMP-PKG signaling pathway was significantly activated in macrophages after 48 h of TPx protein treatment, suggesting its potential involvement in the regulation of macrophage M2 polarization. ELISA detection showed that the cGMP content was significantly increased after 48 h of TPx protein treatment, and Western blot analysis further confirmed that PKG protein expression was significantly upregulated, indicating that the cGMP-PKG signaling pathway plays an important role in TPx protein-induced macrophage M2 polarization. Pretreatment with the PKG inhibitor KT-5823 significantly reversed the TPx-induced upregulation of Arg-1 expression, while having no significant effect on iNOS expression, demonstrating that the cGMP-PKG signaling pathway is an important molecular mechanism by which TPx protein induces macrophage M2 polarization. These findings elucidate the pathogenic mechanism by which *Cysticercus cellulosae* establishes immune evasion through the TPx protein from the perspective of the cGMP-PKG signaling pathway, and provide potential intervention targets for future vaccine development.

Although the present study has identified the cGMP-PKG signaling pathway as a key mediator of TPx-induced macrophage M2 polarization, several limitations should be acknowledged. First, all experiments were performed using THP-1-derived macrophages in vitro, and in vivo studies are needed to confirm these findings. Second, the direct interaction between TPx protein and macrophages has not been explored, and its potential binding partners on macrophages merit further investigation. In addition, the functional roles of *EDNRB*, *NFKBIZ*, and *SOCS1* uncovered by transcriptomic analysis remain to be elucidated.

## 5. Conclusions

The present study demonstrates that *Cysticercus cellulosae* TPx protein induces macrophage M1 polarization at 24 h and promotes M2 polarization at 48 h by activating the cGMP-PKG signaling pathway, representing an important molecular mechanism underlying the immune evasion of *Cysticercus cellulosae*.

## Data Availability

The data that support the findings of this study are available on request from the corresponding author.

## Supplementary Materials

The following supporting information can be downloaded at: https://www.mdpi.com/article/10.3390/pathogens15080843/s1, Figure S1: Morphological observation of PMA-induced differentiation of THP-1 cells into macrophages. (A) THP-1 cells showing suspension growth. (B) Macrophages differentiated by 100 ng/mL PMA stimulation for 48 h, showing adherent morphology; Table S1: Primer sequences used for RT-qPCR; Figure S2: Volcano plot of differentially expressed genes in macrophages treated with TPx protein for 24 h; Figure S3: Gene Ontology (GO) enrichment analysis of differentially expressed genes in macrophages treated with TPx protein for 24 h (top 20 terms); Figure S4: GO enrichment analysis of differentially expressed genes in macrophages treated with TPx protein for 48 h (top 20 terms); Figure S5: KEGG enrichment bubble plot of upregulated pathways in macrophages treated with TPx protein for 24 h; Figure S6: KEGG enrichment bubble plot of downregulated pathways in macrophages treated with TPx protein for 24 h; Figure S7: KEGG enrichment bubble plot of downregulated pathways in macrophages treated with TPx protein for 48 h; Figure S8: Effect of the PKG inhibitor KT-5823 on macrophage viability. Data are presented as mean ± SD (*n* = 3). *** indicates *p* < 0.001, ns indicates *p* > 0.05.
