# Supplementary material for: TPx Protein of Cysticercus cellulosae Regulates Macrophage M2 Polarization via the cGMP-PKG Signaling Pathway
Source: Pathogens. 2026 Aug 13;15(8):843. doi: 10.3390/pathogens15080843 (PMC13516507; doi:10.3390/pathogens15080843)
Supplement: Supplementary file 1 [file pathogens-15-00843-s001.zip › Supplementary Materials_Figure S1–S8.pdf]

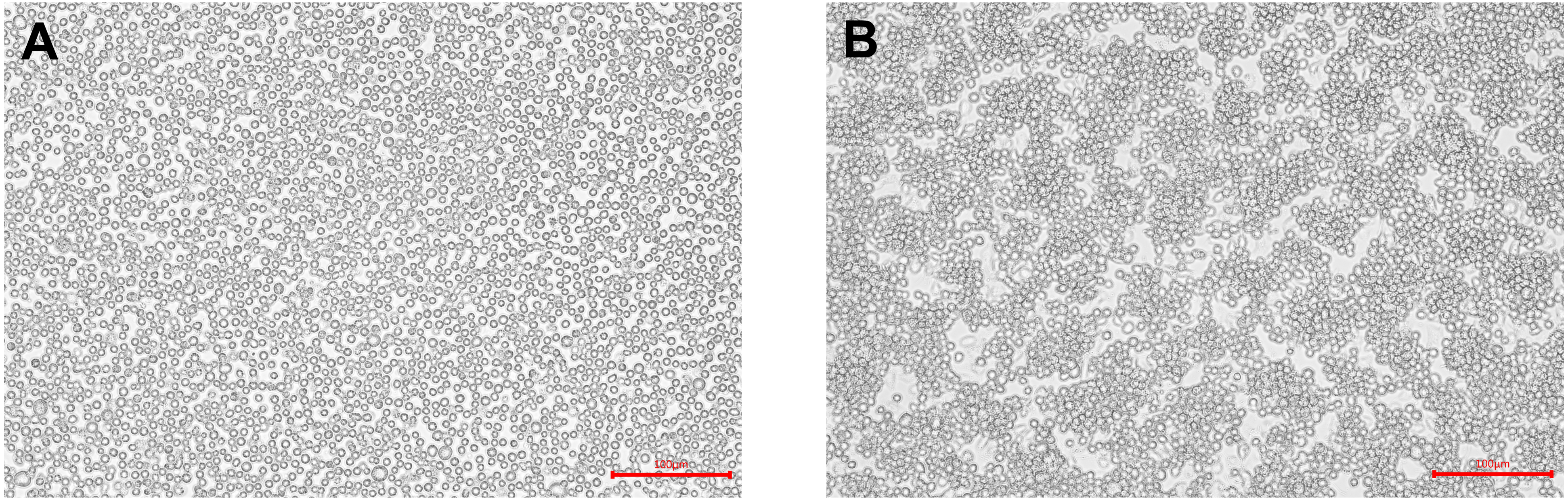

Figure S1. Morphological observation of PMA-induced differentiation of THP-1 cells into macrophages. (A) THP-1 cells showing suspension growth. (B) Macrophages differentiated by 100 ng/mL PMA stimulation for 48 h, showing adherent morphology.

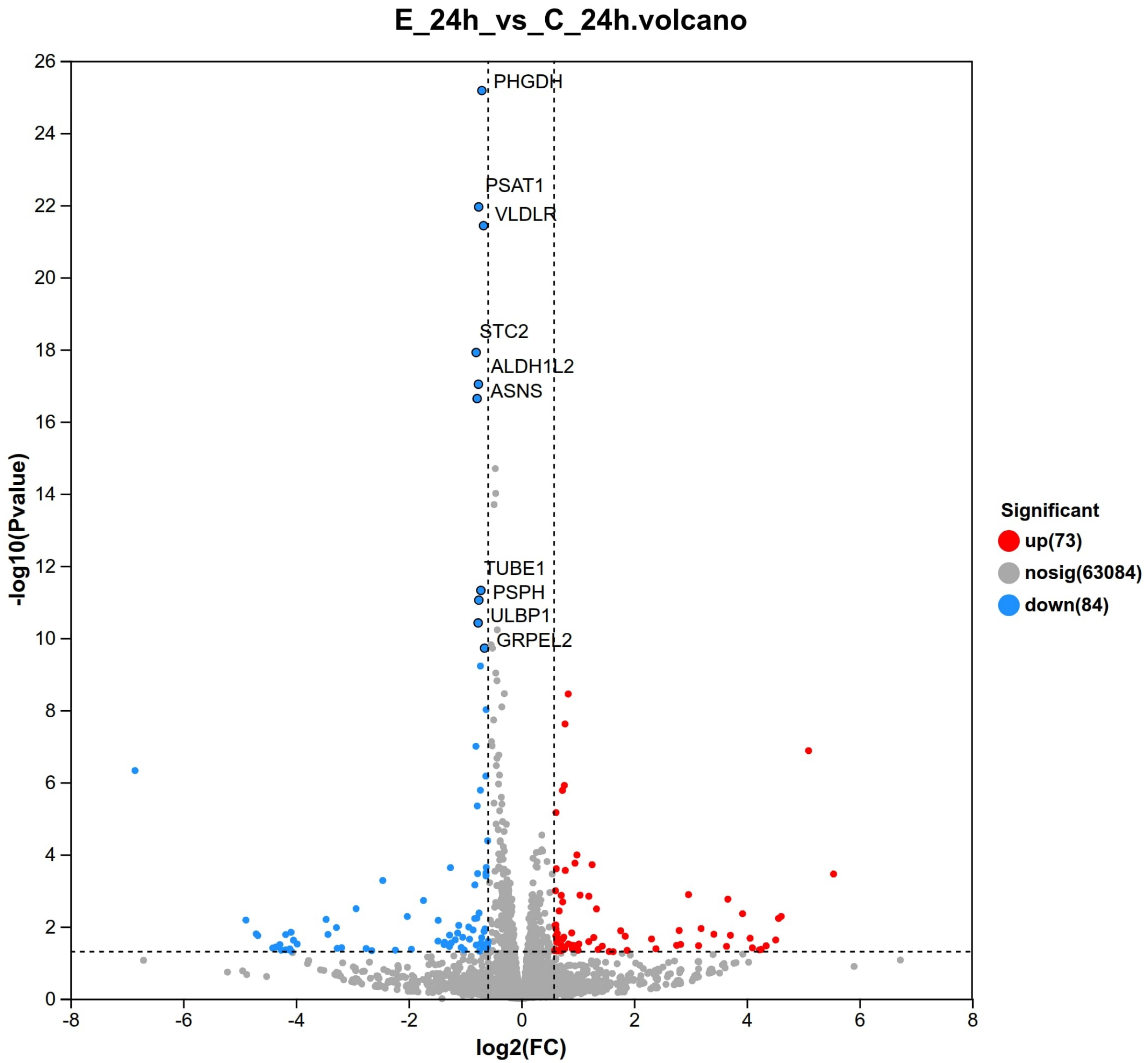

Figure S2. Volcano plot of differentially expressed genes in macrophages treated with TPx protein for 24 h.

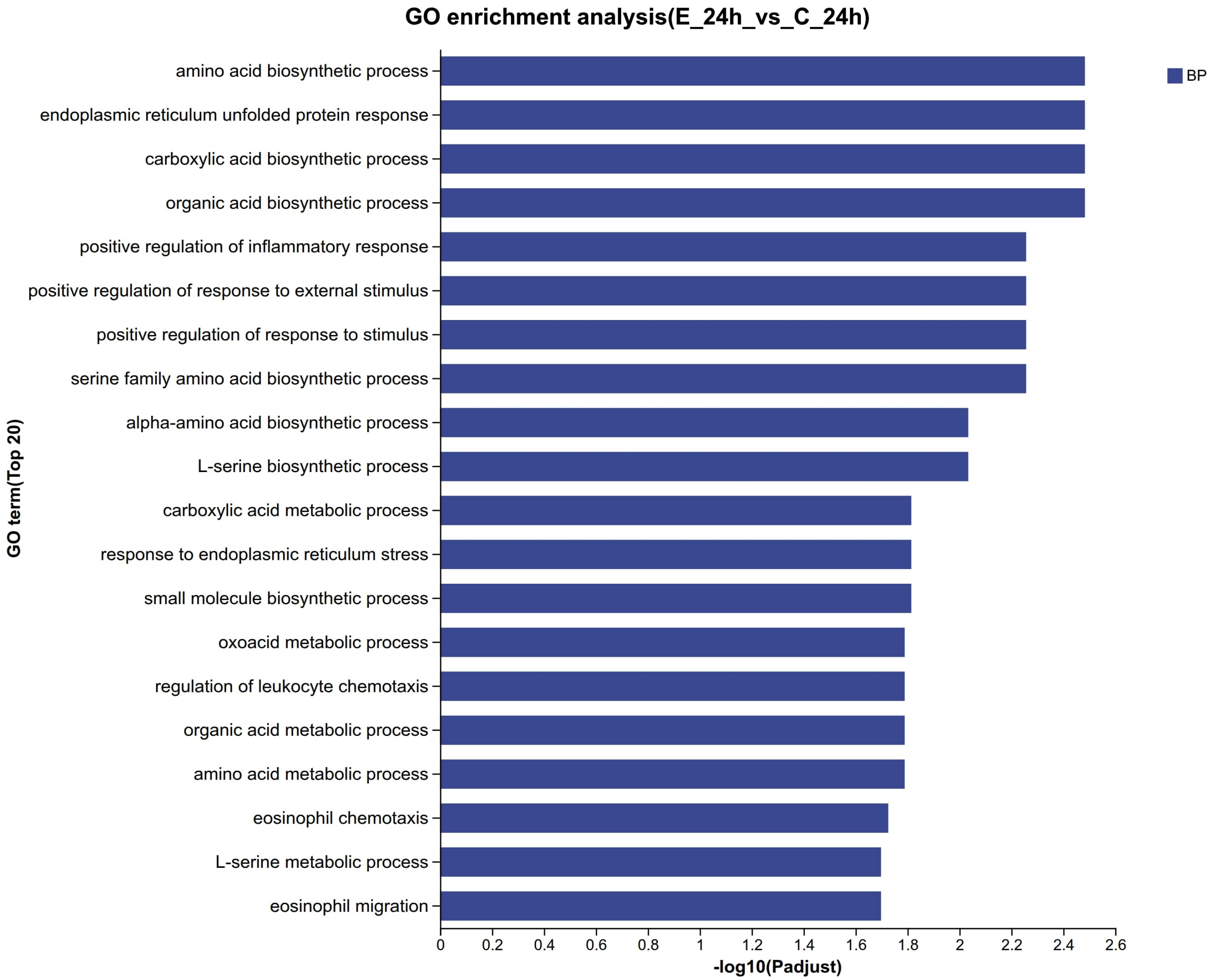

Figure S3. Gene Ontology (GO) enrichment analysis of differentially expressed genes in macrophages treated with TPx protein for 24 h (top 20 terms).

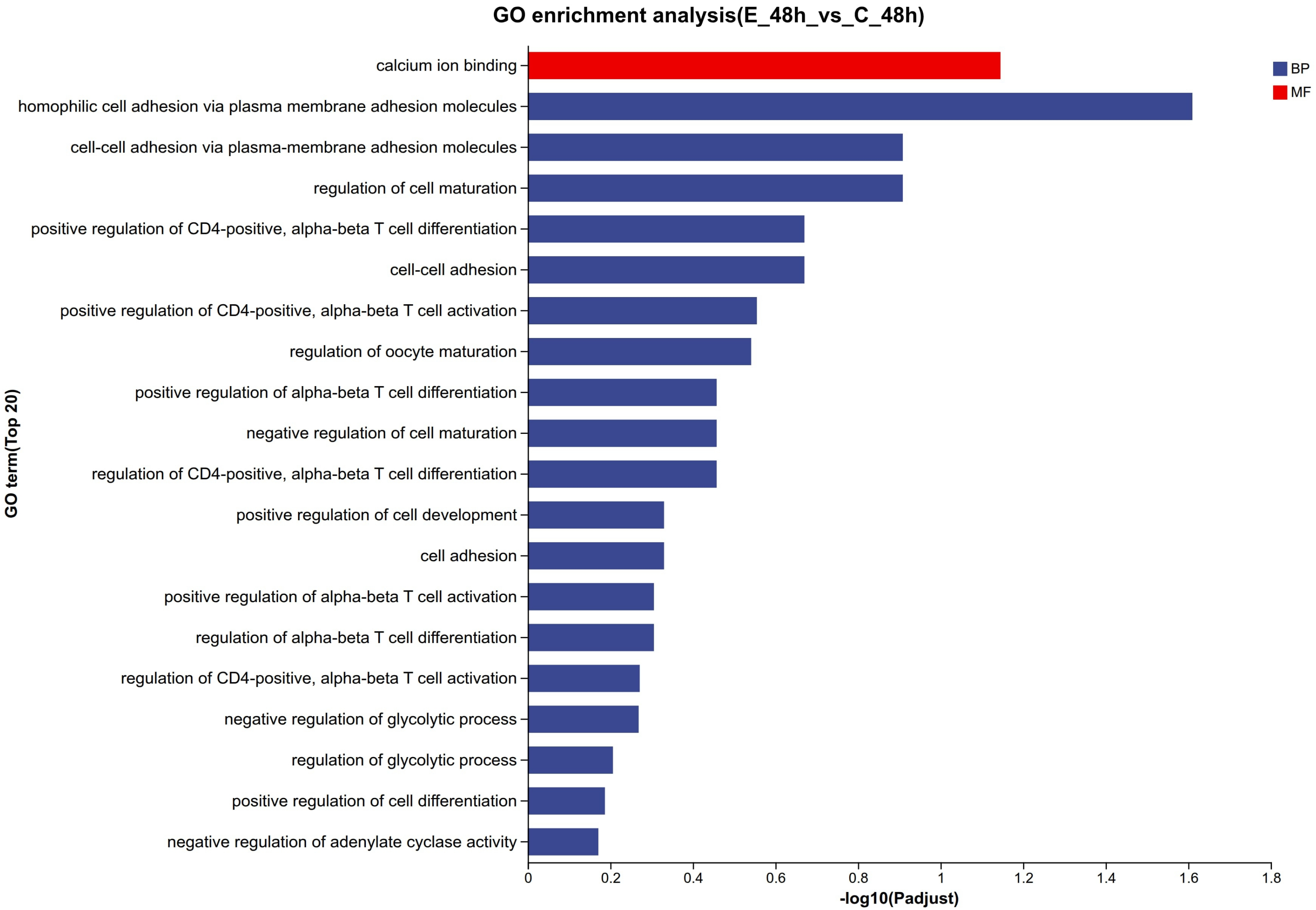

Figure S4. GO enrichment analysis of differentially expressed genes in macrophages treated with TPx protein for 48 h (top 20 terms).

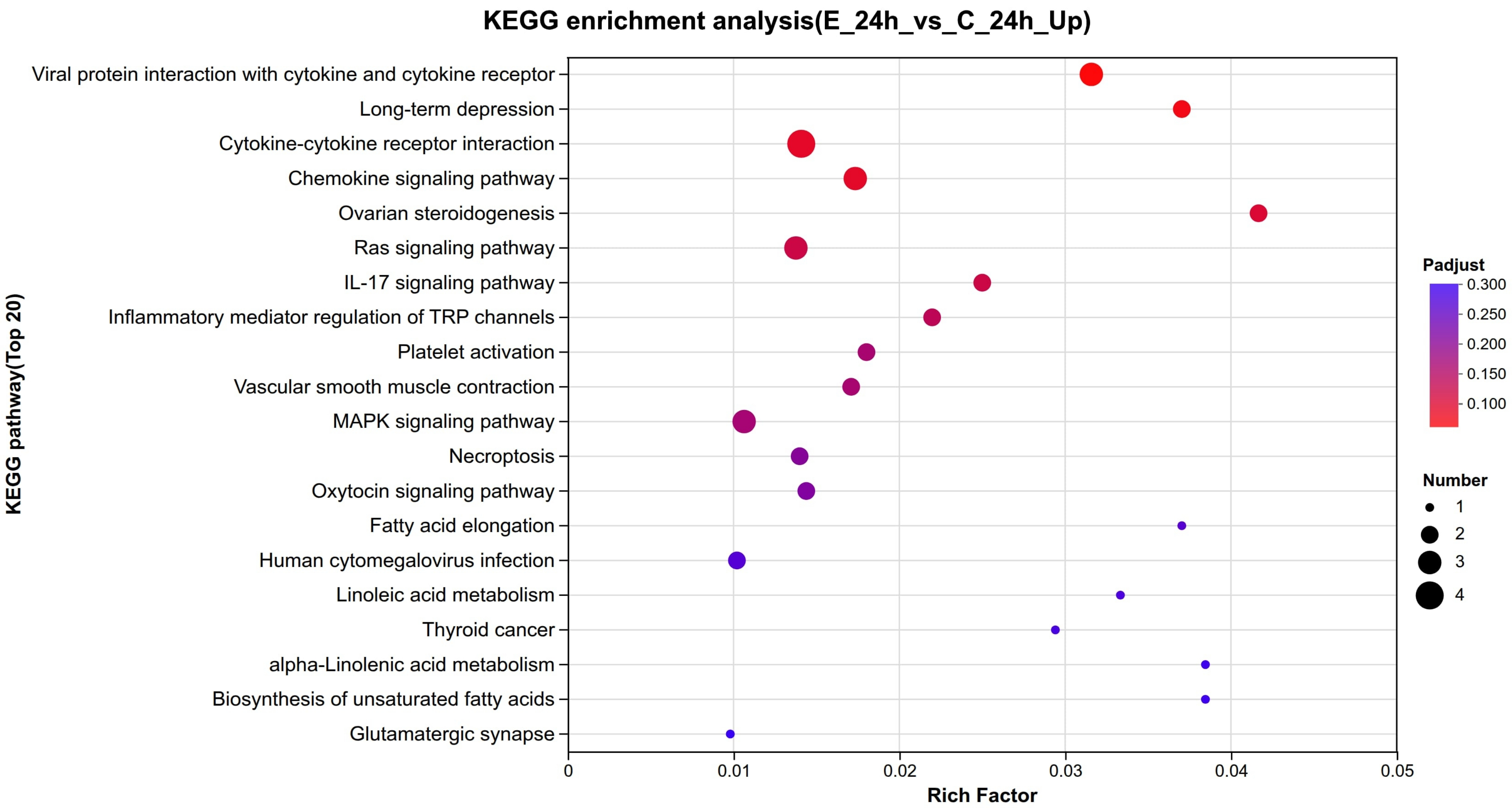

Figure S5. KEGG enrichment bubble plot of upregulated pathways in macrophages treated with TPx protein for 24 h.

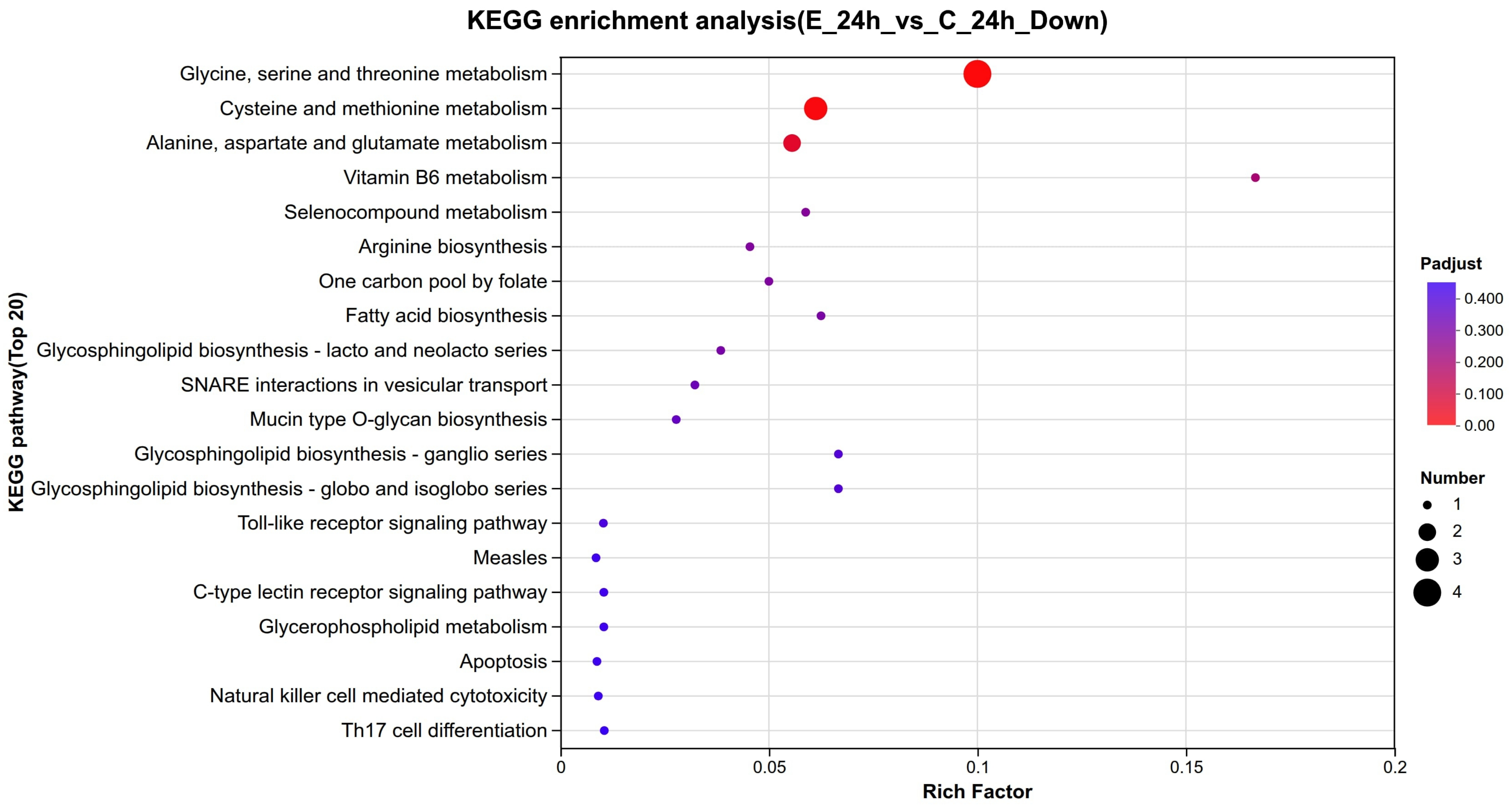

Figure S6. KEGG enrichment bubble plot of downregulated pathways in macrophages treated with TPx protein for 24 h.

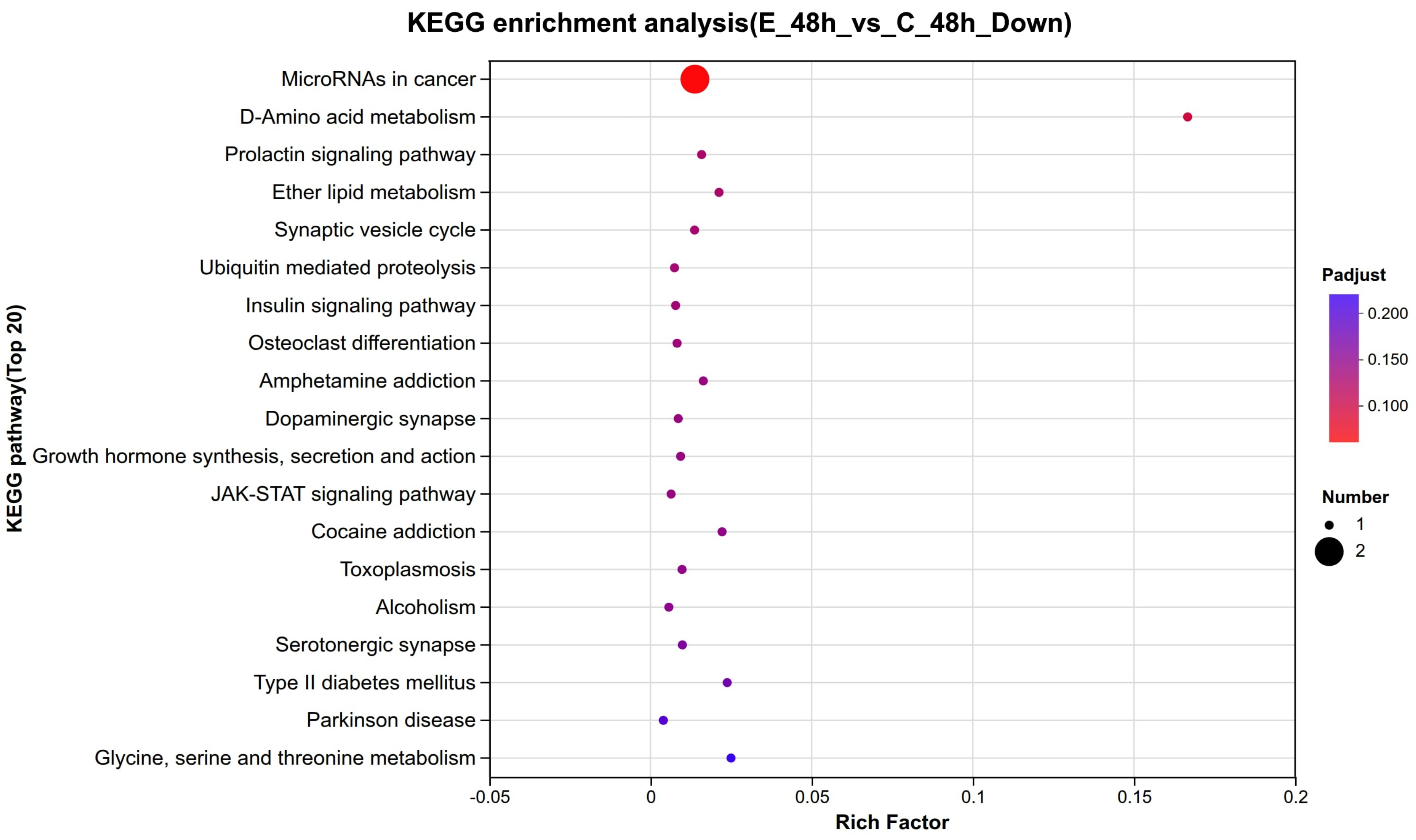

Figure S7. KEGG enrichment bubble plot of downregulated pathways in macrophages treated with TPx protein for 48 h.

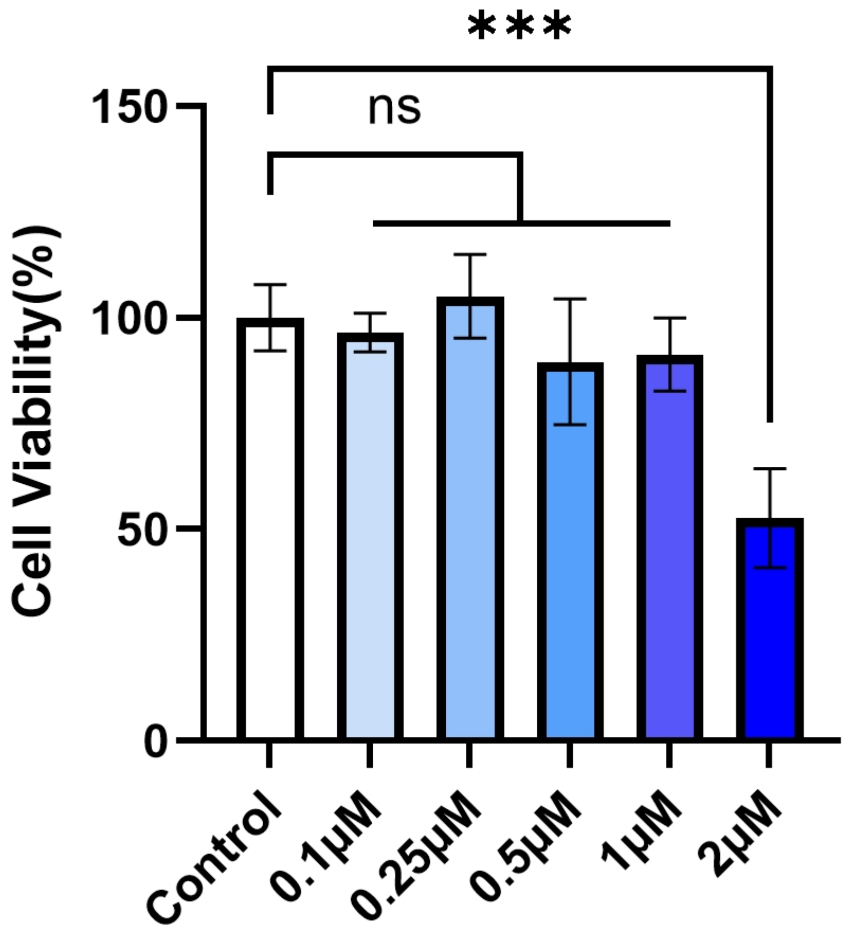

Figure S8. Effect of the PKG inhibitor KT-5823 on macrophage viability. Data are presented as mean ± SD ($n = 3$). *** indicates $p < 0.001$, ns indicates $p > 0.05$.
