# Supplementary material for: TPx Protein of Cysticercus cellulosae Regulates Macrophage M2 Polarization via the cGMP-PKG Signaling Pathway
Source: Pathogens. 2026 Aug 13;15(8):843. doi: 10.3390/pathogens15080843 (PMC13516507; doi:10.3390/pathogens15080843)
Supplement: Supplementary file 1 [file pathogens-15-00843-s001.zip › Supplementary Materials_Table S1.pdf]

Table S1. Primer sequences used for RT-qPCR.

Gene	Forward primer (5'→3')	Reverse primer (5'→3')
TNF-a	CTGTAGCCCATGTTGTAGCAAAC	TTGAAGAGGACCTGGGAGTAGAT
IL-10	AGCTCCAAGAGAAAGGCATCTAC	GTCTATAGAGTCGCCACCCTGAT
GAPDH	GGAGTCCACTGGCGTCTTCA	GTCATGAGTCCTTCCACGATACC
